# A Whole Cell Assay to Measure Caspase-6 Activity by Detecting Cleavage of Lamin A/C

**DOI:** 10.1371/journal.pone.0030376

**Published:** 2012-01-12

**Authors:** Robert Mintzer, Sreemathy Ramaswamy, Kinjalkumar Shah, Rami N. Hannoush, Christine D. Pozniak, Frederick Cohen, Xianrui Zhao, Emile Plise, Joseph W. Lewcock, Christopher E. Heise

**Affiliations:** 1 Department of Biochemical Pharmacology, Genentech, Inc., South San Francisco, California, United States of America; 2 Department of Early Discovery Biochemistry, Genentech, Inc., South San Francisco, California, United States of America; 3 Department of Neuroscience, Genentech, Inc., South San Francisco, California, United States of America; 4 Department of Discovery Chemistry, Genentech, Inc., South San Francisco, California, United States of America; 5 Department of Drug Metabolism and PK, Genentech, Inc., South San Francisco, California, United States of America; Institute of Enzymology of the Hungarian Academy of Science, Hungary

## Abstract

Caspase-6 is a cysteinyl protease implicated in neurodegenerative conditions including Alzheimer's and Huntington's disease making it an attractive target for therapeutic intervention. A greater understanding of the role of caspase-6 in disease has been hampered by a lack of suitable cellular assays capable of specifically detecting caspase-6 activity in an intact cell environment. This is mainly due to the use of commercially available peptide substrates and inhibitors which lack the required specificity to facilitate development of this type of assay. We report here a 384-well whole-cell chemiluminescent ELISA assay that monitors the proteolytic degradation of endogenously expressed lamin A/C during the early stages of caspase-dependent apoptosis. The specificity of lamin A/C proteolysis by caspase-6 was demonstrated against recombinant caspase family members and further confirmed in genetic deletion studies. In the assay, plasma membrane integrity remained intact as assessed by release of lactate dehydrogenase from the intracellular environment and the exclusion of cell impermeable peptide inhibitors, despite the induction of an apoptotic state. The method described here is a robust tool to support drug discovery efforts targeting caspase-6 and is the first reported to specifically monitor endogenous caspase-6 activity in a cellular context.

## Introduction

Caspases are cysteine proteases that mediate a variety of processes including regulation of the inflammatory response and mediating programmed cell death. Various apoptotic caspases have been shown to play a crucial role in embryonic development and tissue homeostasis [1], while deregulation of caspase activity is observed in a variety of pathological conditions. The role that caspase-6 plays in various neurodegenerative conditions is the topic of investigation by numerous groups and highlights the desire to identify selective pharmacological reagents to disrupt enzymatic activity. Several lines of evidence connect caspase-6 with Alzheimer's disease (AD) including localization in disease brains and neurofibrillary tangles [2], [3] as well as direct cleavage of proteins with known involvement in AD progression [4], [5]. Furthermore, axonal degeneration induced by APP activation of DR6 was reported to be mediated by caspase-6 activity [6]. Caspase-6 is also thought to play a role in Huntington's disease as it mediates cleavage of mutant huntingtin protein to induce pathogenesis in relevant disease models [7]–[9]. More recently caspase-6 has also been implicated in Parkinson's disease as the neuroprotective function of DJ-1 protein is dependent on caspase-6 proteolysis [10]. Despite the allure of caspase-6 as a therapeutic target, however, there are no drug-like therapies that selectively modulate this enzyme.

Caspase-6 is classified as an executioner caspase based on its structural homology to caspase-3 and -7 and its requirement for activation by upstream initiator caspases [11], [12], although alternative mechanisms of activation have been proposed [13]. Activated caspase-6 performs proteolytic digestion of a number of substrates with an aspartic acid residue in the P1 position, with P2–P4 amino acid residues conferring substrate specificity against other caspase isoforms [14]. The preferred cleavage motif as defined for caspase-6 is Valine-Glutamate-Isoleucine-Aspartate (VEID) as defined by peptidic substrate mapping [15]. These generalized consensus motifs provide utility as the basis of peptide substrates that are frequently used to interrogate the activity of caspase enzymes. Despite their utility in biochemical assays, there are challenges with enzyme-substrate cross-reactivity [16], [17]. Many of these peptide substrates are processed by a host of different caspase isoforms and would preclude their use in a cellular context where numerous caspase family members are present [17], [18]. The VEID sequence is found at amino acid residues 227–230 in the helical rod region of the nuclear intermediate filament protein lamin A/C. Despite enzyme-substrate cross-reactivity, claims have been made that lamin A/C is proteolyzed only by caspase-6 at this site [19]–[21]. Likewise, many of the available peptide inhibitors have served as useful tools to inhibit enzymatic activity but fail to exhibit selective caspase isoform inhibition [22]. This is likely due to the high degree of active site homology as well as presence of a warhead attached to the inhibitor P1 Asp residue that covalently modifies a conserved catalytic cysteine residue [23]. Peptide inhibitor polarity may also prevent their cell penetration thus precluding their utility as viable tools to assist in the development of cellular caspase assays. It is therefore no surprise that there are no published reports of assays to specifically monitor inhibition of caspase-6 in a cellular context.

Several strategies have been reported to assess pan executioner caspase activity in whole cells including the synthesis of cell penetrant substrates that rely on novel fluorescent dyes or peptide leader sequences to encourage cell uptake [24]–[27]. Another more elaborate strategy to monitor cellular activity of a specific caspase isoform was the utilization of split TEV technology to transiently activate caspase-3. Cellular enzymatic activity from this system [28], or others [29], is readily monitored via engineered FRET reporters. To overcome the hurdles of complex cell engineering and liabilities of possible substrate non-specificity, we developed a whole cell ELISA assay to monitor the proteolysis of endogenously expressed lamin A/C upon induction of the endogenous caspase machinery by apoptosis. We demonstrate that this is a high throughput, robust, whole cell assay for monitoring caspase-6 activity without compromising cellular membrane integrity. The assay serves as a valuable tool for facilitating drug discovery efforts against this important target.

## Materials and Methods

### Inhibitors

Ac-VEID-CHO, Ac-DEVD-CHO, Ac-IETD-CHO, z-VEID-FMK, z-DEVD-FMK, z-VAD-FMK and Q-VD-OPh were obtained from EMD4Biosciences (Gibbstown, NJ). z-VEID-tetrafluorophenoxymethyl (TFPM), z-EID-TFPM and z-ID-TFPM were synthesized by the Discovery Chemistry Department at Genentech, Inc. with procedures described previously [30], [31]. All inhibitor structures can be found in Figure S1.

### Cell Culture

SKNAS Cells (ATCC #CRL-2137) were cultured in DMEM medium supplemented with 10% FBS, 2 µM Glutamax I and 1% Pen/Strep under incubator conditions of 37°Celsius, 90% relative humidity with 5% CO_2_. Cells were split 1∶5 twice weekly. Caspase-6 KO and wild type mouse fibroblasts were cultured under the same medium and conditions. All tissue culture reagents were purchased from Invitrogen (Carlsbad, CA).

### Enzymatic Caspase Activity Assays

Caspases-3, -6 and -7 were expressed in *E. Coli* and purified by the Protein Chemistry Department at Genentech, Inc with procedures similar to those previously published [32]. Biochemical assays of caspase activity were carried out in 384-well black low-volume assay plates (Perkin Elmer #6008260; Waltham, MA) in 12 µl reaction volumes containing enzyme, fluorogenic substrate and inhibitor. All peptide inhibitors were serially diluted in DMSO prior to cross dilution in assay buffer (50 mM HEPES pH 7.2, 25 mM MgSO_4_, 0.5 mM EGTA, 5 mM Glutathione, 0.01% Triton X-100 containing 0.1% Bovine Gamma Globulin (BGG)). Final DMSO concentration in the assay was 1.33%. Rhodamine-labeled bivalent tetrapeptide substrates were obtained from Anaspec (Fremont, CA) and used as follows: 300 pM caspase-3 reacted with 1 µM (Ac-DEVD)_2_ -Rh110, 1 nM caspase-6 reacted with 5 µM (Ac-VEID)_2_ -Rh110, 5 pM caspase-7 reacted with 1 µM (Ac-DEVD)_2_ -Rh110. Enzyme and inhibitor were preincubated for 15 minutes prior to the addition of substrate to initiate the enzymatic reaction. The reaction was permitted to proceed for 40 minutes at room temperature and then read on an Envision (Perkin Elmer) instrument using excitation/emission wavelengths of 485/535 nm. To simplify potency analysis of peptide inhibitors with the capacity to inhibit in a time-dependent manner, the apparent IC_50_'s determined after combined 15 minutes of preincubation and 40 minute enzyme reaction are reported.

### Western Blotting for Cellular Lamin A/C

SKNAS cells were seeded in T-25 flasks (Corning; Lowell, MA) at a density of 5x10^6^ cells/flask in growth medium and allowed to adhere overnight. The following day, staurosporine (Enzo; Plymouth Meeting, PA) was delivered directly to medium in the T-25 flask from a 10 mM stock in DMSO resulting in 3 µM final concentration. Medium was aspirated after 6 hours incubation (or indicated time) followed by addition of 250 µl lysis buffer (Cell Signaling; Beverly, MA). Cell lysates were quantified for protein content using a bicinchoninic acid (BCA) protein assay kit (Thermo; Waltham, MA) and 50 µg of cellular lysate was subjected to SDS-PAGE using 4–12% Bis-Tris gel (Invitrogen) for 90 minutes at 125V. Transfer of protein to 0.45 µm nitrocellulose membrane (Invitrogen) was performed using semi-dry transfer apparatus (BioRad; Hercules, CA) for 60 minutes at 15V. Nitrocellulose membranes were blocked with Odyssey blocking buffer (Licor; Lincoln, NE) for 1 hour at room temperature prior to incubation with 1∶1000 dilution of the indicated primary anti-lamin antibody (rabbit polyclonal targeting a neo-epitope on the small subunit of cleaved lamin A/C (Cell Signaling #2035S); rabbit polyclonal targeting a neo-epitope on the large subunit of cleaved lamin A/C (Cell Signaling #2031S); rabbit polyclonal targeting intact lamin A/C (Cell Signaling #2032)) or 1∶5000 dilution of mouse monoclonal anti-β actin antibody (Sigma #A5441) for 60 minutes at room temperature with gentle rocking motion. The membrane was then washed 4 times for 5 minutes with Tris Buffered Saline containing 0.05% Tween-20 (TBST) (Invitrogen) followed by incubation with secondary antibody for 1 hour at room temperature. Dilutions of 1∶2500 (HRP-conjugated donkey anti-rabbit IgG (GE Healthcare #NA934V; Piscataway, NJ)), 1∶10000 (IRDye 800 conjugated affinity purified anti-mouse IgG (Rockland, Gilbertsville, PA)) and 1∶2000 (Alexa Fluor 680 goat anti-rabbit IgG (Invitrogen, Carlsbad, CA)) were used. Finally, the membrane was washed 4 times for 5 minutes in TBST and scanned using a LiCor Odyssey Infrared Scanning instrument (LiCor; Lincoln, NE).

### Cell-based ELISA for Cleaved Lamin

SKNAS or mouse fibroblast (wild type and caspase-6 KO) cells were plated at a density of 20,000 cells per well into poly-D-lysine coated 384-well black clear-bottom cell culture plates (Becton Dickinson #35-4663; Franklin Lakes, NJ) in growth medium and allowed to adhere overnight. The following day, cell culture medium was aspirated and replaced with 20 µl/well basal medium (DMEM with no supplements added). All peptide inhibitors were serially diluted in DMSO prior to cross dilution in basal medium. 5 µl of diluted inhibitor was delivered to each well and incubated for 30 minutes in a cell culture incubator prior to addition of 5 µl/well staurosporine in basal medium. The final DMSO concentration in the assay was 0.1% and the final staurosporine concentration was 3 µM (or indicated concentration). Following 6 hours incubation with staurosporine, 10 µl of 16% paraformaldehyde (Alfa Aesar #43368; Ward Hill, MA) was added directly to each well and incubated at room temperature for 15 minutes before careful aspiration of each well and two 100 µl washes with Phosphate Buffered Saline (PBS) (Invitrogen). Cells were permeabilized by the addition of 50 µl 0.2% Triton X-100 in PBS for 15 minutes at room temperature. Each well was aspirated, washed twice with 100 µl PBS, and nonspecific binding sites blocked with 100 µl blocking agent (Thermo #37535) for 10 minutes at room temperature. The blocking agent was replaced with 20 µl/well rabbit polyclonal primary antibody (Cell Signaling #2035S) delivered at 1∶200 dilution in blocking agent and incubated overnight at 4°Celsius. The following day, primary antibody was aspirated and the wells washed 3 times with 100 µl TBST. Secondary antibody (donkey anti-rabbit IgG, HRP-conjugated (GE Healthcare #NA934V)) was delivered at 1∶2500 dilution in blocking agent (20 µl/well) and incubated 1 hour at room temperature. Following aspiration, the plate was washed 5 times with 100 µl/well TBST, chemiluminescent HRP substrate (Thermo #37074) was added and luminescence measured 5 minutes later using a Perkin Elmer Envision instrument.

### *In vitro* Lamin A cleavage assay

Active human recombinant caspase-6 was provided by the Protein Chemistry Department at Genentech, Inc. All other active caspases were purchased from Enzo LifeSciences (Cat # BML-AK010) and used at a final concentration of 5 U/µl (300U total; see manufacturer's guide for description). In each assay, recombinant glutathione S-transferase **(**GST)-tagged lamin A (17 µg/ml, 170 nM) (Abnova Cat# H00004000-P01; Taipei City, Taiwan) was incubated with active caspase enzyme at 37°C for 2 hours in assay buffer (50 mM HEPES (pH 7.5), 100 mM NaCl, 1 mM EDTA, 0.5% CHAPS, 10 mM DTT). For caspase-9, the assay buffer was: 50 mM HEPES (pH 7.5), 100 mM NaCl, 0.01% CHAPS, 0.5 M NaCitrate, 1 mM EDTA, 10 mM DTT. Samples were incubated at 100°C. for 5 minutes, resolved by SDS-PAGE (12% Tris-glycine, Invitrogen) and transferred to a nitrocellulose membrane. The blot was probed with horseradish peroxidase (HRP)-conjugated anti-GST antibody (1∶1000) (Sigma-Aldrich; St. Louis, MO). The signal was developed with Chemi-IR™ detection kit using IRDye 800CW rabbit anti-HRP antibody (1∶1000) (Licor) according to manufacturer's recommendation. The membrane was scanned on a LiCor Odyssey Infrared Scanning instrument. Under the same assay conditions, all enzymes were verified in fluorogenic assays to be active in processing their specific tetrapeptide-AMC substrates (data not shown).

The biochemical cleavage assay of lamin A with increasing concentrations of human active caspase-6 (as indicated) was done in 40 mM HEPES (pH 7.2), 100 mM NaCl, 0.1% CHAPS, 80 µM DTT for 2 hours at 37°C. The samples were processed as described above and the signal was quantified using Odyssey software.

### Caspase-6 Knockout Mouse

The construct for targeting the C57BL/6 caspase-6 locus in ES cells was made using a combination of recombineering [33], [34] as well as standard molecular cloning techniques.

Briefly, a 6537 bp fragment (assembly NCBI37/mm9, chr3∶129,606,653-129,613,189) from a C57BL/6 mouse BAC (RP23-233B14) was first retrieved into plasmid pBlight-TK [34]. Second a loxP-em7-kanamycin-loxP cassette was inserted upstream of exon 2 between position chr3∶129,608,133 and129,608,134. Correctly targeted plasmid was transformed into arabinose-induced SW106 cells (Cre expressing E.coli, [35], [36]) to remove kanamycin and leave behind a single loxP site. Finally, an frt-PGK-em7-Neo-BGHpA-frt-loxP cassette was inserted downstream of exon 4 between position chr3∶129,609,697 and 129,609,698, resulting in the caspase-6 conditional knock-out (CKO) targeting vector. The final vector was confirmed by DNA sequencing.

The caspase-6 CKO vector was linearized with NotI and C57BL/6 C2 ES cells were targeted using standard methods (G418 positive and gancyclovir negative selection). Positive clones were identified using PCR and taqman analysis, and confirmed by sequencing of the modified locus. Correctly targeted ES cells were transfected with a Flpe plasmid to remove Neo and create the caspase-6 conditional knock-out allele (CKO), or with a Cre plasmid to remove Neo and create the caspase-6 KO allele. Caspase-6 KO or CKO ES cells were then injected into blastocysts using standard techniques, and germline transmission was obtained after crossing resulting chimaeras with C57BL/6N females.

### Data Analysis

The endpoint absorbance, fluorescent (RFU) or luminescent (RLU) emission in each well was plotted as a function of inhibitor concentration and the 50% inhibition (IC_50_) values were determined using a nonlinear least squares fit of the data to a four parameter equation using Prism 5.0 software (GraphPad Software, San Diego, CA).

### Caspase-Glo® 6 Assay

SKNAS cells were plated at a density of 20,000 cells per well into 384-well black clear-bottom cell culture plates in 20 µl/well growth medium and allowed to adhere overnight. The following day, 5 µl of peptide inhibitor diluted in growth medium was delivered to each well 30 minutes prior to addition of 5 µl/well staurosporine in growth medium. The final DMSO concentration in the assay was 0.1% and the final staurosporine concentration was 3 µM. Following 6 hours incubation at 37°C, Caspase-Glo® 6 reagents (Promega; Madison, WI) were prepared according to manufacturer's recommendation, and 30 µl was added directly to each well, followed by 1 hour incubation at 37°C and luminescence recording using a Perkin Elmer Envision.

### LDH Release Assay

SKNAS cells were plated at a density of 20,000 cells per well into 384-well black clear-bottom cell culture plates in 20 µl/well growth medium and allowed to adhere overnight. The following day, 5 µl staurosporine diluted in growth medium was delivered to each well and incubated for the indicated time in a cell culture incubator. The final DMSO concentration in the assay was 0.1%. At each timepoint, lactate dehydrogenase (LDH) activity was measured in cell supernatants using a colorimetric LDH activity measurement kit (BioVision; Mountain View, CA) according to manufacturer's recommendation by measuring absorbance at 450 nm using a Spectramax platereader (Molecular Devices; Sunnyvale, CA).

### Mass Spectrometry Analysis of Intracellular Inhibitor Accumulation

SKNAS cells were seeded in 12-well plates at a concentration of 5×10^6^ cells/mL and allowed to adhere overnight prior to the experiment. Caspase inhibitors (10 µM) in Hank's balance Salt Solution (HBSS; Invitrogen) buffer containing 10 mM HEPES (Invitrogen) and a final DMSO concentration of 0.1% were added in triplicate to the cells. Non specific binding (NSB) samples were prepared by immediately aspirating the dose solution and washing the cells with 1 mL of ice cold HBSS. Cells for the accumulation experiment were incubated for 1 hour at 37°C with inhibitors prior to washing with 1 mL ice cold HBSS. Analytical internal standard (200 nM propranolol (Sigma) in 100% acetonitrile (ACN) with 0.1% formic acid) and blank HBSS buffer were sequentially added to the 12-well plates containing the NSB and accumulation samples. Reference samples for each inhibitor were made by adding internal standard to dose solution and cells incubated for 1 hour at 37°C. The cells from all experiments were scraped from the bottom of the 12 well plates, sonicated for approximately 30 minutes, and centrifuged for 10 minutes at 3000 x g prior to LC/MS/MS analysis.

The LC/MS/MS system consisted of an AB Sciex API4000 mass spectrometer (AB Sciex, Foster City, CA), an Aria LX2 multiplexing LC system (Thermo Fisher Scientific, Waltham, MA) and a CTC Analytics autosampler (Leap Technologies, Carrboro, NC). An ESI Spray source in the positive ion mode and multiple reaction monitoring (MRM) were used to quantitate peak areas. The LC separations were performed on a 50×2.0 mm Gemini C18 analytical column (Phenomenex, Torrance, CA). The mobile phases consisted of water with 0.1% formic acid (mobile phase A) and ACN with 0.1% formic acid (mobile phase B). Mass spectrometer data were processed using Analyst software version 1.4.2.

## Results

### Peptide based inhibitors and substrates lack isoform selectivity

There are a wide number of commercially available pharmacologic reagents that can be used to inhibit the enzymatic activity of recombinant caspases. Most of these tools are peptide inhibitors based on preferred substrate recognition sequences that possess an electrophilic warhead coupled to the P1 Aspartic acid residue for direct interaction with the enzyme's catalytic cysteine. For example, VEID-aldehyde (Ac-VEID-CHO) is generally inferred as selectively inhibiting caspase-6 while Ac-DEVD-CHO is considered a caspase-3/7 inhibitor. In order to elucidate the ability of these tools to dissect executioner caspase activity in a cellular context, a panel of peptide based caspase inhibitors with divergent amino sequences and warheads was tested for their ability to inhibit the enzymatic activity of caspase-3, -6 and -7 (Table 1). Under the assay conditions tested, aldehyde-containing peptide inhibitors were the most potent against caspase-6 (relative to fluoro-methyl ketone (FMK) derived inhibitors), but the amino acid sequence appears to confer little selectivity. For example, Ac-DEVD-CHO (IC_50_ = 30.5 nM) and Ac-IETD-CHO (IC_50_ = 53.2 nM), which are canonically considered selective for caspase-3 and caspase-8, respectively, inhibited caspase-6 with near equal potency as did Ac-VEID-CHO (IC_50_ = 16.2 nM). This lack of selective inhibition by these tools was seen for each of the executioner caspases tested (Table 1). As an extension to this notion, we monitored the proteolysis of a distinct fluorescent substrate with a VEID recognition motif ((Ac-VEID)2-Rh110) by each of the three executioner caspases. This substrate was readily cleaved under initial rate conditions by caspase-3 and -7 with efficiency comparable to caspase-6 (data not shown) confirming previous findings with similar peptide substrates [17], [18]. This illustrates the limitation with using any of these reagents as tools to selectivity monitor caspase-6 activity in a cellular context where other executioner caspases are present.

**Table 1:** Potency of peptide-derived caspase inhibitors possessing aldehyde (CHO) and fluoromethyl ketone (FMK) warheads against executioner caspases.

	Mean IC_50_ * (nM)		
Sample	Caspase-6	Caspase-3	Caspase-7
Ac-VEID-CHO	16.2	13.6	162.1
Ac-DEVD-CHO	30.5	1.3	3.7
Ac-IETD-CHO	53.2	75.8	552.8
z-VEID-FMK	128.6	45.1	648.0
z-DEVD-FMK	402.3	35.8	98.5
z-VAD-FMK	2112	168.0	74.7

*IC_50_ values were determined after a 15 minute enzyme/inhibitor preincubation and 40 minute enzyme reaction. Due to the capacity for time-dependent inhibition with irreversible inhibitors the values reported can be considered “apparent IC_50_”.

### Lamin processing by caspase-6

**Figure 1:**
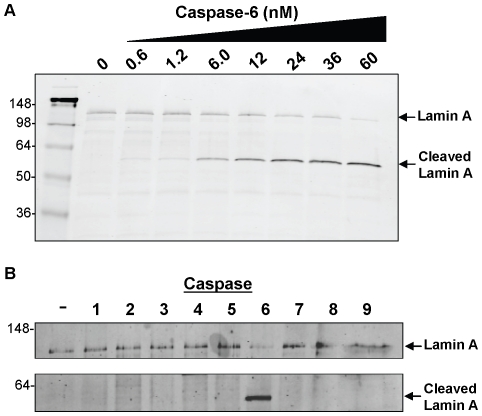
Western blot detection of recombinant GST-lamin A processing by purified caspases. (A) The indicated concentration of caspase-6 was incubated with GST-lamin A for two hours. (B) 300 Units of caspases 1–9 were incubated with GST-lamin A for two hours. Intact and cleaved lamin A were detected via western blotting using anti-GST antibody.

Due to the lack of selectivity of peptide reagents listed above, we sought to identify a full length substrate that might offer a higher degree of selective processing. The nuclear envelope proteins lamin A and lamin C have previously been identified as substrates for caspase-6 and contain the consensus VEID sequence in a helical rod section between N and C terminal globular domains. These two proteins are encoded by a single primary transcript and therefore share the identical first 566 amino acids with the only differences occurring in the C-terminus [37]. The efficiency of purified active caspase-6 to process recombinant lamin A was evaluated by monitoring its degradation over a range of enzyme concentrations (Figure 1A). Lamin degradation is evident with as little as 6 nM caspase-6 (30 U).

To assess proteolytic specificity, the capacity of other caspase isoforms to degrade recombinant lamin A was evaluated. Western blot analysis of the reaction products from 300 U active caspases 1–9 reveals that only caspase-6 produced a prominent signal for cleaved lamin (Figure 1B). It was observed that at higher caspase-3 concentrations (>1000 U) slight lamin processing does occur (data not shown), although these enzyme conditions are non-catalytic. Each enzyme was separately evaluated in independent enzymatic experiments to ensure proficient activity and found to possess suitable activity and proper pharmacology (data not shown).

### Development of cellular lamin A/C degradation assay

**Figure 2:**
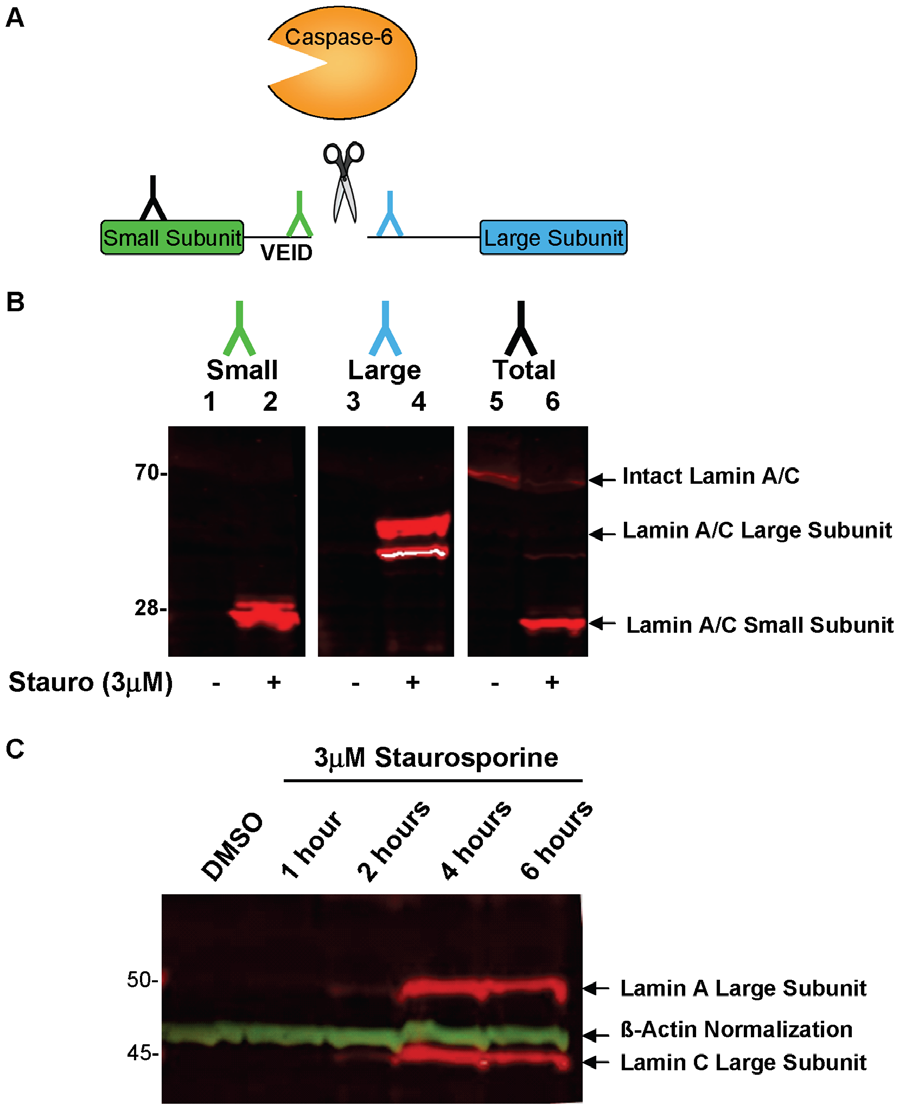
Western blot detection of lamin A/C from SKNAS neuroblastoma cells upon staurosporine treatment. (A) Schematic of the N- and C-terminal globular domains of Lamin A/C with VEID-containing central α-helical region as the site of caspase-6 proteolysis. (B) SKNAS cells were treated with DMSO control or staurosporine for 6 hours prior to cell lysis. Lysates were probed for small lamin A/C subunit (Lanes 1–2), large lamin A/C subunit (Lanes 3–4) or total lamin A/C (Lanes 5–6). (C) SKNAS cells were treated with DMSO or staurosporine for the indicated time prior to cell lysis. Lysates were probed for large lamin A/C subunit (red) or β-Actin (green).

The high degree of selective lamin proteolysis by recombinant caspase-6 prompted us to investigate the use of this substrate as a cellular readout for caspase-6 activity. Staurosporine is a non-selective protein kinase inhibitor that activates caspase-6 [38], amongst other caspases, in cells as they progress towards apoptosis. Cell lysates were prepared from SKNAS cells treated with DMSO or 3 µM staurosporine for six hours to activate the caspase machinery. The SKNAS cell line is of human neuroblastoma origin, and was selected for these and following studies due to its robust caspase induction as assessed using Caspase-Glo® 6 assay kit from Promega as well as by observing zymogen conversion to active caspase-6 by western blot detection (data not shown). A panel of three antibodies that bind intact lamin or its proteolytic fragments were monitored for their ability to detect each lamin A/C species via western blot (Figure 2A and 2B). All three antibody preparations detected intact lamin A/C (Lane 5) or its large (Lane 4) or small (Lane 2) proteolytic fragments. As seen in lane 6, the intact lamin A/C antibody also detects the small proteolytic fragment produced upon staurosporine treatment suggesting its binding epitope is on the N-terminus of the protein. On the other hand, the antibodies against the large or small subunit recognize neo-epitopes only exposed subsequent to caspase-6 proteolysis and do not detect intact lamin A/C (Lanes 1, 3). The kinetics of lamin A/C degradation were monitored by inducing an apoptotic state with 3 µM staurosporine for 1–6 hours and identified the abundant appearance of proteolytic fragments after 4–6 hours of staurosporine treatment (Figure 2C). Induction of cellular caspase activity with 6 hours of staurosporine treatment was also observed using the Caspase-Glo® 6 assay kit (data not shown) and provided confidence that this time point was suitable for the remainder of our assay development.

**Figure 3:**
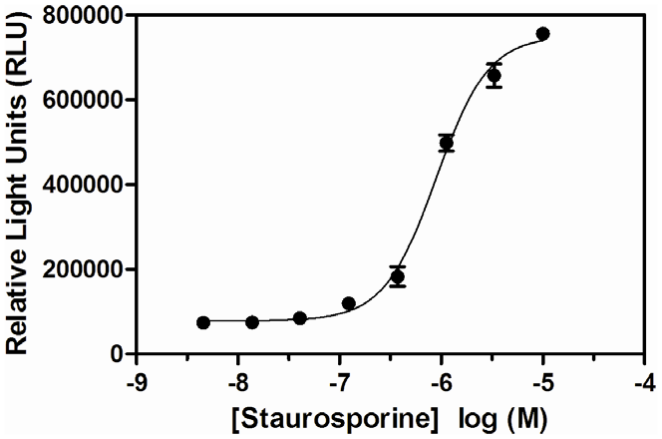
Effect of staurosporine on the generation of cleaved lamin A/C in SKNAS cells. SKNAS cells were treated with the indicated concentration of staurosporine for 6 hours prior to detection of the small lamin A/C cleavage product as described in Experimental Procedures. The assay was performed in triplicate one time. The mean and standard error of the mean are reported.

In order to develop an assay more amenable to inhibitor screening, this antibody detection was adapted to a plate-based non-denaturing assay. SKNAS cells cultured in 384-well plates were treated with 3 µM staurosporine for six hours prior to fixation with 4% paraformaldehyde and permeabilization with 0.2% Triton X-100. Binding of the two antibodies that recognize neo-epitopes on the large or small lamin degradation products was detected by horseradish-peroxidase-labeled secondary antibody and subsequent chemiluminescent substrate addition (see methods for details). In this context, the large subunit antibody was unable to produce a signal window (data not shown), while the small subunit antibody generated a robust signal and was utilized for further studies. The concentration dependence of staurosporine required to evoke lamin A/C degradation after a six-hour treatment period indicated that 3 µM staurosporine produced a marked signal:background ratio of 8.8-fold and that higher concentrations could be used to induce a greater response if desired (Figure 3). Under these conditions the assay proved highly robust (Z'≥0.7).

**Figure 4:**
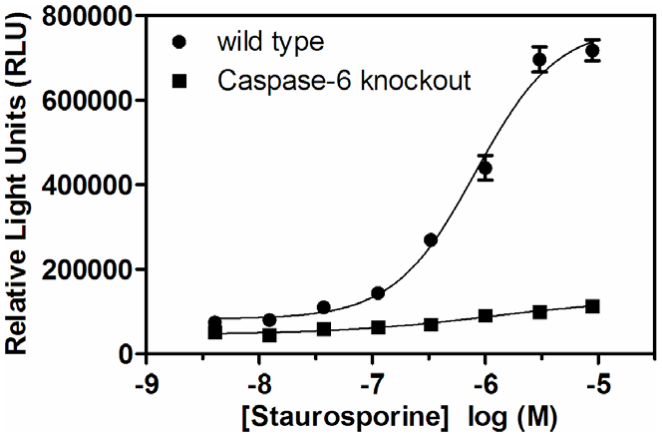
Apoptosis-mediated cleavage of lamin A/C is elevated in wild-type relative to caspase-6 KO fibroblasts. Fibroblasts derived from caspase-6 KO (▪) or wild type (•) mice were treated with the indicated concentration of staurosporine for 6 hours prior to detection of the small lamin A/C cleavage product. The assay was performed in quadruplicate two times with similar results; mean and standard error of the mean are reported.

This lamin degradation plate-based assay was used to further investigate the specificity of lamin A/C as a caspase-6 substrate in a cellular environment. Murine embryonic fibroblast cultures were established from tail-tissue-explants derived from caspase-6 knockout and wild-type mice and subjected to increasing concentrations of staurosporine prior to detection of cleaved lamin (Figure 4). The marked response in the wild-type cells compared to the caspase-6 knockout cells supports the biochemical evidence that caspase-6 is the primary caspase responsible for lamin processing, although emergence of lamin degradation products begin to appear slightly at the highest staurosporine concentration tested in the caspase-6 KO fibroblast cells. Under these conditions, the morphological features associated with apoptosis were equally observed in both the wild type and KO cells (data not shown) as would be expected given the ability of staurosporine to function as a universal apoptotic trigger with known capacity to activate the caspase-3 machinery [27], [39].

### Membrane integrity is not compromised during caspase activation

**Figure 5:**
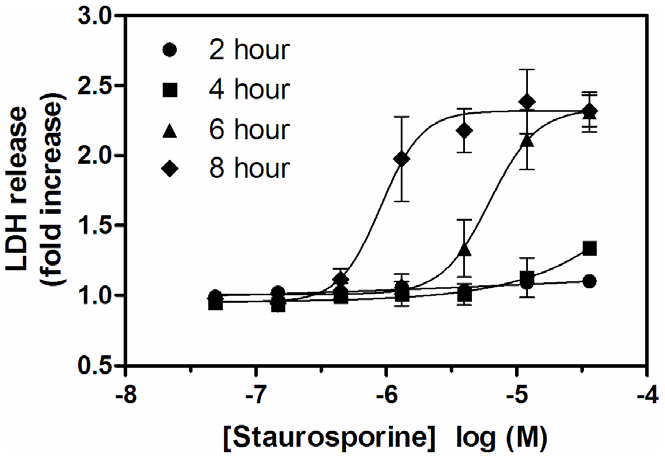
Effect of staurosporine on the release of Lactate Dehydrogenase in SKNAS cells. SKNAS cells were treated with the indicated concentration of staurosporine for 2 (•), 4 (▪), 6 (▴) or 8 (♦) hours prior to detection of LDH release to the cell supernatant. The assay was performed in triplicate and represents 1 of at least 2 experiments with similar results. The data was normalized to fold increase over DMSO treatment. The mean and standard error of the mean are reported.

The goal of the cell-based assay is to measure inhibition of the intracellular caspase-6 by cell-permeant inhibitors, therefore it is important to establish that cell membrane integrity has not been compromised during caspase activation. To assess the integrity of the SKNAS cell membrane under the staurosporine-induced apoptotic conditions, lactate dehydrogenase (LDH) release was measured upon treatment of cells with a range of staurosporine concentrations and after several incubation time periods. As seen in Figure 5, the concentration dependent release of LDH in these cells is also highly dependent on the incubation time. After 6 hours of treatment, LDH release is detected above background levels with ∼4 µM staurosporine. The same degree of measured LDH release with 30 µM staurosporine only requires 4 hours of treatment. These data also illustrate that under conditions in which maximal lamin cleavage was obtained by wild type but not caspase-6 KO fibroblasts, cytotoxicity as measured by LDH release was minimal. This information can facilitate the selection by the user of incubation time and staurosporine concentration to develop their preferred assay.

### Peptide inhibitor pharmacology in lamin A/C cleavage assay

**Figure 6:**
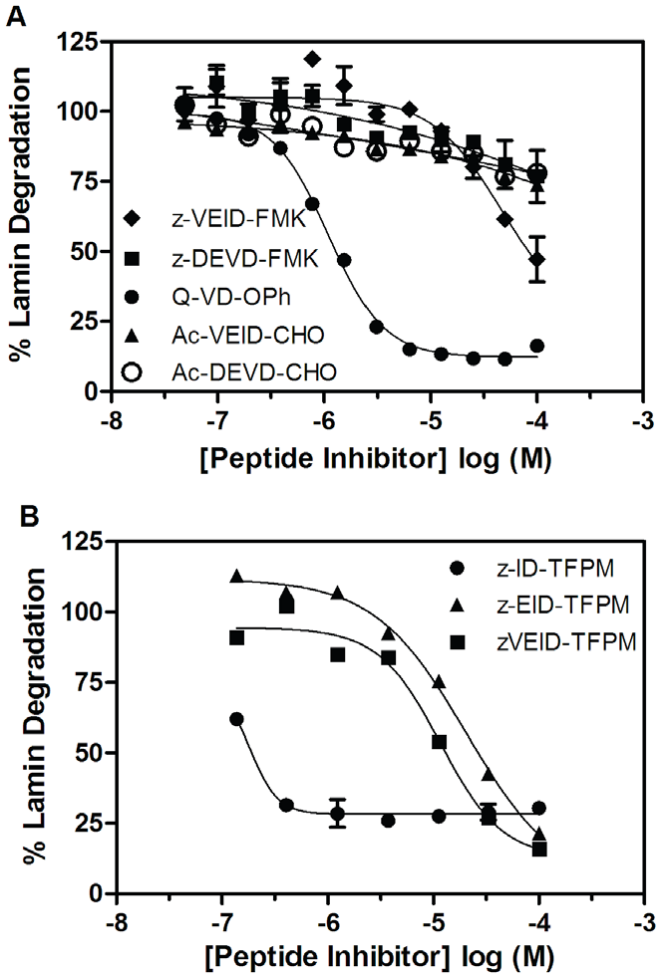
Effect of peptide-based caspase inhibitors on the generation of cleaved lamin A/C in SKNAS cells. (A) SKNAS cells were treated with z-VEID-FMK (♦), z-DEVD-FMK (▪), Q-VD-OPh (•), Ac-VEID-CHO (▴) or Ac-DEVD-CHO (○) prior to addition of 3 µM staurosporine for 6 hours. (B) SKNAS cells were treated with z-ID-TFPM (•), z-EID-TFPM (▴) or z-VEID-TFPM (▪) prior to addition of 3 µM staurosporine for 6 hours. Detection of the small lamin A/C cleavage product was performed as described in Experimental Procedures. Concentration inhibition curves were performed in duplicate and represent 1 of at least 3 experiments with similar results. Concentration-response curves for each inhibitor were normalized to zero and 100% based on no staurosporine or DMSO, respectively. The mean and standard error of the mean are reported.

**Figure 7:**
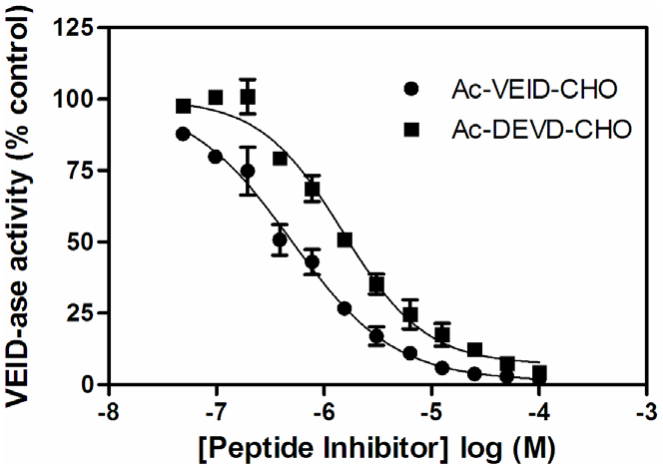
Effect of peptide-based caspase inhibitors on SKNAS cells as determined using the Caspase-Glo® 6 assay. SKNAS cells were treated with Ac-VEID-CHO (•) or Ac-DEVD-CHO (▪) prior to addition of 3 µM staurosporine for 6 hours and detection of VEID-ase activity as described in Experimental Procedures. Concentration inhibition curves were performed in duplicate and represent 1 of at least 3 experiments with similar results. Concentration-response curves for each inhibitor were normalized to zero and 100% based on no staurosporine or DMSO, respectively. The mean and standard error of the mean are reported.

The plate-based version of the lamin degradation assay was used to monitor the potency of a panel of peptide inhibitors for their capacity to prevent caspase-6-dependent proteolysis. At inhibitor concentrations up to 100 µM, little or no inhibitory activity was observed with the aldehyde or FMK based tetrapeptide inhibitors possessing a Glu at the second position despite having submicromolar biochemical potencies (Figure 6A and data not shown). The nonselective inhibitor z-VAD-FMK inhibited with 44 µM potency consistent with previously published reports [40]. This result might be expected given the highly charged nature of these Glu-containing molecules which prevents their cellular accumulation. Conversely, the pan caspase inhibitor Q-VD-OPh, which possesses a difluorophenoxymethyl warhead, inhibited generation of the small lamin subunit with an IC_50_ of 940 nM (6-fold lower potency than enzymatic assay) (Figure 6A, Table 2). To this end, three further peptide inhibitors were synthesized each with a similar warhead (tetrafluorophenoxymethyl (TFPM)) with the attempt to facilitate cell activity (Figure S1). These new inhibitors are based off the z-VEID recognition motif and are successively truncated at the N-terminus (z-VEID-TFPM, z-EID-TFPM, z-ID-TFPM). In biochemical caspase-6 assays, each truncation results in a decrease in potency (1.7nM, 8.4nM, 19nM), while the opposite is seen in the cellular assay (Figure 6B, Table 2). In this case, the shortest and least charged inhibitor z-ID-TFPM inhibited lamin degradation most potently with an IC_50_ = 180nM (∼10-fold less potent than enzymatic inhibition). In order to confirm our hypothesis that lack of cellular activity of these inhibitors is due to their poor membrane permeability, we analyzed the degree of their SKNAS cellular accumulation via mass spectrometry. This analysis revealed that cellular potency is highly correlative with cell penetrance. Q-VD-OPh and z-ID-TFPM were quantified as the two inhibitors most readily able to penetrate into cells and also the two most effective at inhibiting lamin degradation (Table 2). Ac-VEID-CHO is predominantly excluded from accessing the intracellular environment (0.16% cellular accumulation) and lacks any activity in the cellular assay. When the membrane barrier is removed as in the case of the lysate based Caspase-Glo® 6 assay, this peptide inhibitor as well as Ac-DEVD-CHO (also inactive in lamin degradation assay) are both clearly able to inhibit VEIDase activity (Ac-VEID-CHO IC_50_ = 0.49 µM; Ac-DEVD-CHO IC_50_ = 1.49 µM) (Figure 7). It is evident from these data that inhibition in the lamin degradation assay is dictated by a compound's absolute binding affinity and its ability to access the intracellular target.

**Table 2:** Comparison of potency of peptide-derived caspase inhibitors in cellular lamin cleavage assay, enzymatic activity and cell permeability.

Sample	Cellular Lamin Cleavage IC_50_ (µM)	Enzymatic Caspase-6 IC_50_ *(µM)	% cell accumulation
Ac-VEID-CHO	>100	0.016	0.16
Ac-DEVD-CHO	>100	0.031	Nd
z-VEID-FMK	45	0.129	0.46
z-DEVD-FMK	>100	0.402	nd
z-VAD-FMK	44	2.11	nd
Q-VD-OPh	0.94	0.156	1.72
z-VEID-TFPM	12	<0.0017^a^	0.17
z-EID-TFPM	21	0.0084	nd
z-ID-TFPM	0.18	0.019	1.97

nd = not determined

a = z-VEID-TFPM enzymatic IC_50_ is less than 0.0017 due to limit of enzymatic assay detection.

*Enzymatic IC_50_ values were determined after a 15 minute enzyme/inhibitor preincubation and 40 minute enzyme reaction.

## Discussion

Caspase-6 is an important drug target that is implicated in various neurodegenerative diseases, yet the availability of tools to monitor caspase-6 activity in a cellular context is limited. Lysate based assays such as the Caspase-Glo® 6 kit from Promega function as useful tools to monitor general caspase activity but the value of this assay as a tool for drug discovery to assess inhibitor permeability into cells is diminished because of the required lysis step. Additionally, peptide substrates of this type have been shown to be readily processed by multiple caspase isoforms and may not offer the desired specificity to support drug discovery [18]. Furthermore, peptide inhibitors lack the required selectivity profile to shut down specific branches of the caspase signaling cascade that is activated in an apoptotic setting [22]. Given the limitations of these tools, we developed a cellular assay to monitor caspase-6 activity by detecting the proteolytic digestion of a specific substrate, lamin A/C to allow interrogation of selective caspase-6 inhibitors.

Lamin A/C is processed by caspase-6 after Asp230 to generate small and large proteolytic digestion products. We utilized the availability of antibodies that recognize neo epitopes accessible only upon cleavage to develop an ELISA assay to monitor generation of the 25 KDa small lamin product. In order to activate the caspase machinery we took advantage of the largely cytotoxic effect of staurosporine on SKNAS neuroblastoma cells to invoke an apoptotic state. The time and concentration dependent induction of lamin degradation and LDH release by staurosporine illustrated within this manuscript provide a detailed indication of the state of cell health as well as a framework for optimization of assay parameters required by other investigators. The assay itself proved very robust with a capacity to support large scale chemistry efforts directed against caspase-6. The strong assay window is likely due to utilization of neo-epitope antibodies that do not detect any product formation in the absence of staurosporine treatment combined with signal amplification conferred by the ELISA assay platform.

In order to confirm the validity of this assay as a useful tool to interrogate cellular caspase-6 activity, we performed a series of experiments to address the concern of selectivity. Only purified caspase-6 was able to effectively proteolyze recombinant lamin A compared to the extensive panel of caspase isoforms tested. It is likely that the VEID consensus motif presented in the context of a full-length protein is able to confer a degree of specificity not afforded with peptide substrates. It is also possible that secondary interactions outside the consensus site contribute to its specificity. Furthermore, lamin A/C degradation was markedly reduced in fibroblasts extracted from caspase-6 knock-out mice relative to fibroblasts from wild type mice. The source of the small amount of remaining lamin A/C cleavage in these KO fibroblasts at the highest staurosporine concentration might represent crossreactivity of other caspase isoforms, particulary given the fidelity of caspase-3 and caspase-7 for their ability to process VEID containing substrates, or perhaps unrelated proteases capable of cleaving this protein such as Granzyme A [41]. It is evident from the KO data combined with biochemical specificity data, however, that cleavage of lamin A/C is a sound strategy as a readout for caspase-6 activity in cells.

This assay was pharmacologically evaluated by profiling a panel of peptide inhibitors for their ability to prevent lamin A/C degradation. Surprisingly, only inhibitors with warheads capable of covalent cysteine modification (fluoromethylketone, difluorophenoxymethyl and tetrafluorophenoxymethyl) were able to prevent the cleavage of lamin A/C while aldehyde-based inhibitors lacked cell activity. Although this analysis is confused by the low permeability of the aldehyde inhibitors, this observation might provide suggestive evidence that covalent modification of caspase-6 is a prerequisite for in vivo efficacy. Extensive experimentation beyond the scope of this manuscript is required to completely address this notion. The strong disconnect between cellular and biochemical potency for many of the inhibitors was easily rationalized by measuring the intracellular accumulation of the peptides whose highly charged nature might contribute to cell exclusion. These data serve to assist in the pharmacological validation of this assay as well as provide direct evidence that despite cells existing in an apoptotic state, they maintain cell membrane integrity. Overall, the assay will aid in the discovery of selective caspase-6 inhibitors with reasonable physiochemical properties.

In summary, we report here a whole-cell high throughput 384-well assay which monitors cellular caspase-6 cleavage of endogenous lamin A/C under apoptotic conditions. The assay is performed in a physiologically relevant cell line without the necessity of genetic engineering and is able to discriminate against chemical compounds with poor membrane penetrance. This assay is the first reported to monitor caspase-6 activity in the context of mammalian cells and the capacity of the antibody to react across multiple species enhances the versatility of this technique for adaptation to a variety of cell backgrounds. With recent data implicating caspase-6 as an important mediator of neurodegenerative diseases, the assay described here enables drug discovery efforts to characterize cell-active inhibitors of this target.

## Supporting Information

Figure S1 Chemical structures of peptide inhibitors possessing aldehyde (CHO), Fluoromethyl Ketone (FMK), Difluorophenoxymethyl (OPh) or Tetrafluorophenoxymethyl (TFPM) warheads.
(TIF)
